# Seasonal Dynamics in the Chemistry and Structure of the Fat Bodies of Bumblebee Queens

**DOI:** 10.1371/journal.pone.0142261

**Published:** 2015-11-11

**Authors:** Alena Votavová, Aleš Tomčala, Edita Kofroňová, Michaela Kudzejová, Jan Šobotník, Pavel Jiroš, Olga Komzáková, Irena Valterová

**Affiliations:** 1 Agricultural Research, Ltd., Troubsko, Czech Republic; 2 Institute of Organic Chemistry and Biochemistry, Academy of Sciences of the Czech Republic, Prague, Czech Republic; 3 Institute of Parasitology, Biology Centre, Czech Academy of Sciences, České Budějovice, Czech Republic; 4 Faculty of Forestry and Wood Sciences, Czech University of Life Sciences, Prague, Czech Republic; University of Graz, AUSTRIA

## Abstract

Insects’ fat bodies are responsible for nutrient storage and for a significant part of intermediary metabolism. Thus, it can be expected that the structure and content of the fat body will adaptively change, if an insect is going through different life stages. Bumblebee queens belong to such insects as they dramatically change their physiology several times over their lives in relation to their solitary overwintering, independent colony foundation stage, and during the colony life-cycle ending in the senescent stage. Here, we report on changes in the ultrastructure and lipid composition of the peripheral fat body of *Bombus terrestris* queens in relation to seasonal changes in the queens’ activity. Six life stages are defined and evaluated in particular: pharate, callow, before and after hibernation, egg-laying, and senescence. Transmission electron microscopy revealed that the fat body contained two main cell types–adipocytes and oenocytes. Only adipocytes reveal important changes related to the life phase, and mostly the ration between inclusion and cytoplasm volume varies among particular stages. Both electron microscopy and chemical analyses of lipids highlighted seasonal variability in the quantity of the stored lipids, which peaked prior to hibernation. Triacylglycerols appeared to be the main energy source during hibernation, while the amount of glycogen before and after hibernation remained unchanged. In addition, we observed that the representation of some fatty acids within the triacylglycerols change during the queen’s life. Last but not least, we show that fat body cell membranes do not undergo substantial changes concerning phospholipid composition in relation to overwintering. This finding supports the hypothesis that the cold-adaptation strategy of bumblebee queens is more likely to be based on polyol accumulation than on the restructuring of lipid membranes.

## Introduction

Bumblebees (Hymenoptera: Apidae: Bombini) are pollinators of prime importance in moderate climates. This feature has long been exploited by humans, and bumblebees are responsible for a significant increase in the quality and quantity of harvests both in greenhouses and in the field [1,2].

A bumblebee colony is an annual phenomenon that is established by a sole queen in the spring and dies off during the autumn. The queen has the longest life of all colony members, and this can be divided into several phases that differ considerably in terms of the queen’s activities, energy and environmental requirements, and utilization of fat body (FB) reserves [3]. The queen spends the first days of her adult life in the natal nest and later (July to August) leaves it in order to copulate with usually a single unrelated male, chosen based on the quality of the sexual and arrestment signal produced in the labial glands [4–7]. Only a fraction of young fertilized queens survive the winter, and their success is often linked to the amount of energy stored in their FB [8–10]. In spring, the queen first restores her energy reserves and then begins laying eggs and nurturing her first offspring. She stops collecting food after the first workers have emerged and remains within the nest for the rest of her life. In *Bombus terrestris*, the production of a new generation of reproductives starts approximately 4 weeks after the first workers’ emergence, and the nest founder usually dies during this period [11].

Knowledge as to the composition of bumblebee energy reserves consists in only a few studies, reporting namely quantifications of glycogen content in freshly emerged queens and workers [12] and male triacylglycerols (TG) profile in several bumblebee species [13–15]. Unlike short-lived males with a single dominant activity, queens are exposed to many energy-demanding processes. We hypothesized that the queen’s FB, as a major energy storage organ and a site of intermediary metabolism, undergoes extreme changes in structure and function over the course of the queen’s life, with lipid accumulation before and after hibernation, lipid consumption during hibernation and colony founding, and adaptation to low temperatures [16–19]. Cold adaptation runs through two basic mechanisms, i.e. polyol accumulation and/or restructuring of membrane phospholipids (PL). A similar adaptation may occur in bumblebees. Thus, we hypothesized that bumblebees’ cell membranes restructure in response to cold during overwintering.

To the best of our knowledge, none of the above hypotheses have been verified nor have the phenomena been studied in detail. Therefore, we focused on studying structural changes in the FB as well as changes in lipid composition [intact TG, PL, fatty acids (FA)] in six life stages of *B*. *terrestris* queens, namely in pharate, freshly emerged, pre-hibernating, post-hibernating, young, and senescent queens.

## Materials and Methods

### Biological material

*Bombus terrestris terrestris* (Linnaeus, 1758) queens (buff-tailed bumblebee) originated from laboratory cultures at the Research Institute for Fodder Crops, Ltd. Troubsko, Czech Republic [20]. The laboratory conditions of nest development simulated a natural photoperiod. The studied queens (198 specimens) were used in the following stages: pharate queens (immature individuals black in color extracted from pupal cuticles shortly before emerging), callow queens (the first day of adult stage), mated queens before hibernation (age 5 days), queens during hibernation (age 15 weeks, 3 weeks at 28°C, and 3 months at 4°C; only for membrane PL investigation), queens after hibernation (after 5 months at 4°C), egg-laying queens (5th day after the first eggs were laid or on the day when the first cocoon of the brood spooned into cocoons), and senescent queens (from colonies already producing their own sexuals).

### Microscopy analyses

Two living queens of each age were subjected to structural study. These were submerged into fixative (a mixture of 2% glutaraldehyde and 2.5% formaldehyde in 0.1 M phosphate buffer), and several pieces of abdominal tergites and sternites (together with the underlying FB) were cut out with fine scissors. Further steps were taken similarly to those in a previously published protocol [5].

To evaluate adipocyte size, three sections per sample were analyzed and each originated at least 50 μm (100 sections) from other studied semi-thin sections of the same sample. We determined the three largest adipocyte sections, because these probably represent the real size of original cells, and measured the length and width of each. Adipocyte shape was considered to be ellipsoid and its volume was calculated from two measured cell dimensions; the third was approximated as the mean of the other two. To evaluate the proportion of each particular inclusion type (lipids, glycogen, and proteins), transmission electron microscopy (TEM) images were analyzed quantitatively by manually evaluating each kind of inclusion according to the specific characteristic of each: proteins appear as heavy electron-dense biocrystals or granules, lipids as moderately electron-dense droplets located freely in the cytoplasm, and glycogen in the form of small berry-like rosettes. Other lipid classes (diacylglycerols, PL, etc.) were not included into the semi-quantification because identifying these analytes is beyond the capability of the TEM methodology. ImageJ software (National Institutes of Health, USA, public domain http://www.scijava.org/) was used to count up the selected areas. Cumulative areas were expressed as a percent of the total adipocyte section and at least 10,000 μm^2^ of adipocytes sections were analyzed per sample (between 20,000 and 35,000 μm^2^ per stage).

### Chemicals

Standards of TG (purity 99%) were purchased from Larodan Fine Chemicals (Malmö, Sweden) and Nu-Chek Prep (Elysian, MN, USA). Gradient grade Acetonitril-CHROMASOLV^®^ for high-performance liquid chromatography (HPLC) (Sigma-Aldrich, St. Louis, MO, USA) as well as gradient grade methanol and *n*-hexane (Merck, Kenilworth, NJ, USA) were used as received; other solvents (chloroform, diethyl ether, and 2-propanol) were distilled in glass from analytical-grade products (Penta, Prague, Czech Republic). 2,6-Di-*tert*-butyl-4-methylphenol (butylated hydroxytoluene) and ammonium acetate were purchased from Fluka (Buchs, Switzerland).

### Sample preparation

Queens were frozen at −18°C and their abdomens were then cut off. Within 24 h, the FB were dissected by scraping from the inner side of the abdominal cuticle, extracted in chloroform:methanol (1:1,v/v), and treated as previously reported [21]. FB extracts were evaporated to dryness and total lipids were weighed. The remainder of the body (head, thorax, and legs) was dried for 2 h at 80°C followed by 1 h at 110°C and then weighed.

Several replications (5–7 specimens) of each life stage were used for TG analyses. Samples were stored at −80°C prior to analyses. Crude extracts were separated using thin-layer chromatography and the TG fractions were treated as previously described [13]. For the study of cold adaptation, two groups of queens (n = 5) were used: 1) queens after mating and prepared for hibernation (pre-overwintering), and 2) queens during overwintering (at 4°C for 3 months) in laboratory conditions. FB were dissected and extracted directly as the queens were taken from the refrigerator.

### High performance liquid chromatography/mass spectrometry (HPLC/MS)

HPLC/MS with atmospheric pressure chemical ionization (APCI) was used for the TG analyses as described by Jiroš et al. [15]. In order to evaluate the total lipid composition, we used HPLC combined with electrospray ionization mass-spectrometry (MS-ESI) (modified according to ref. 19). This method is especially useful for studying membrane PL and their changes during overwintering. Lipids were separated on a Gemini HPLC column (150 × 2.0 mm, 3 μm; Phenomenex, Torrance, CA, USA). The mobile phase was composed of (A) ammonium acetate in methanol (5 mmol/L), (B) water, and (C) 2-propanol. A linear gradient of A:B:C changing from 92:8:0 to 50:0:50 within 80 min was used with a flow rate of 150 μL/min. The mass spectrometer was operated with ESI source in the positive ion detection mode at +4 kV and capillary temperature at 220°C. Nitrogen was used as both the shielding and the auxiliary gas. Mass range 440–1100 Da was scanned every 0.5 s to obtain the ESI mass spectra. For the investigation of glycerolipid structures, the collision-induced decomposition multi-stage ion trap tandem spectra MS^2^ were recorded with respective 5 and 3 Da isolation windows. Maximum ion injection time was 100 ms and collision energies were 30% (MS^2^).

### Statistical evaluations

To evaluate differences in adipocyte size among the queen stages, the log-transformed data were subjected to One-way ANOVA with Tukey's post-hoc multiple comparison test performed using R package, Version 2.14.2 (http://www.R-project.org/). The data obtained from the TEM analysis (glycogen, lipid droplets and protein granules), as well as TG and PL peak areas were statistically evaluated using ordination methods as follows: for all data–detrended correspondence analysis (DCA); for linear data–principal component analysis (PCA), redundancy analysis (RDA), and Monte-Carlo permutation test (unrestricted permutations, n = 999); and for unimodal data–correspondence analysis (CA), constrained correspondence analysis (CCA), and Monte-Carlo permutation test. For each, the relative inclusion areas and peak areas were calculated in the deconvoluted total cell and peak area. In the canonical analyses (RDA, PCA), the queens’ age, or stage, stood as a categorical predictor. Monte-Carlo permutation tests were used to determine statistical significance. CANOCO 4.5 statistical software (Biometris, Plant Research International, Wageningen UR, Netherlands) was used for the DCA, CA, CCA, PCA, RDA, and Monte-Carlo permutation test analyses.

## Results

The tables and figures referred to as S1–S6 (S1–S5 Figs, S1–S6 Tables) in the following text are available in Supporting Information. Raw data for each sample (PL analytical data, TG analytical data, FA recalculations, adipocyte size measurements and calculations, weights of total lipids, TG, and bodies without abdomens) are given in S1 Dataset in the Supporting information.

### Fat body structure

**Fig 1:**
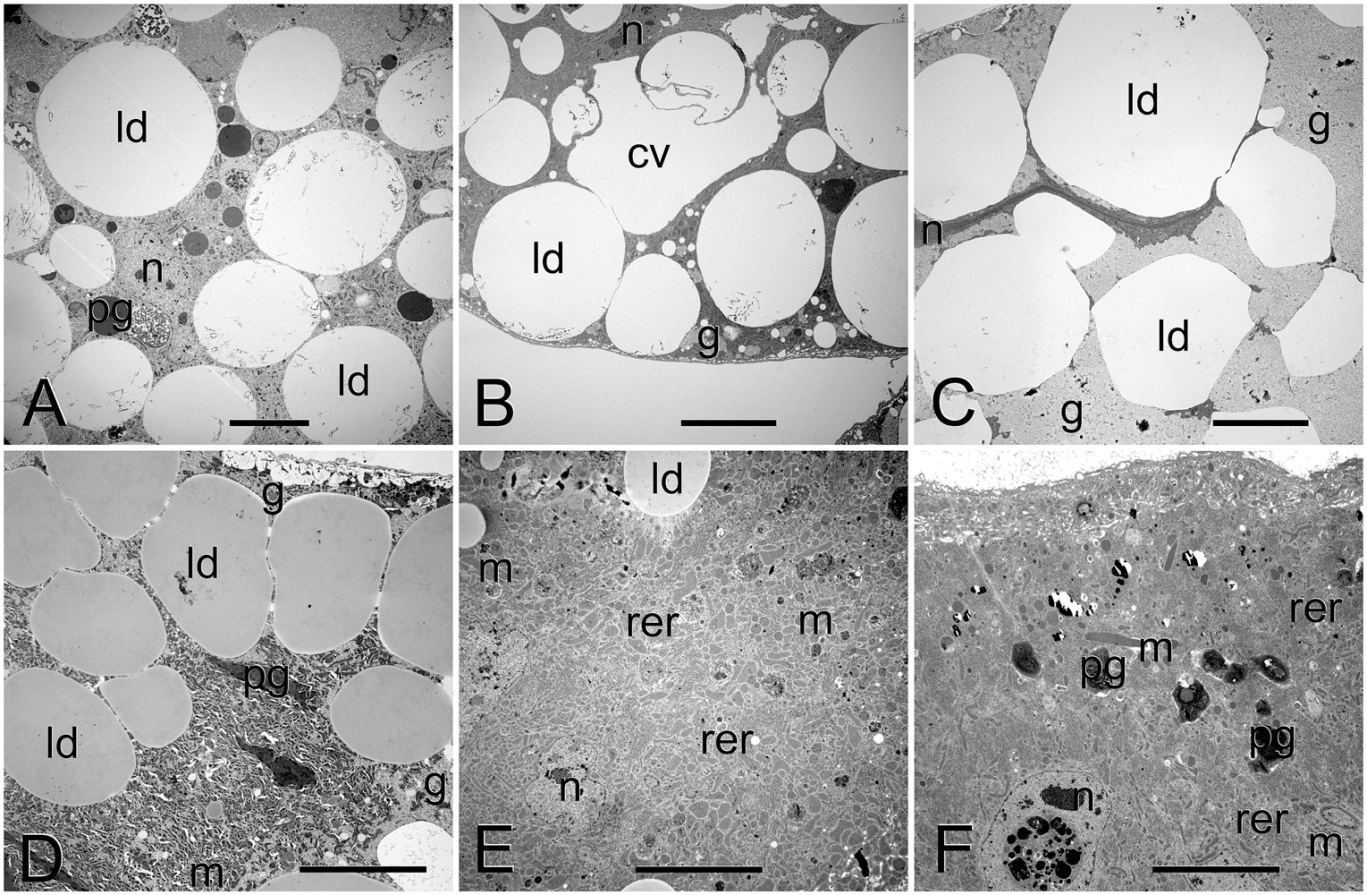
Ultrastructure of adipocytes in *B*. *terrestris* queens: pharate (A), callow (B), before hibernation (C), after hibernation (D), egg-laying (E), and senescent (F). Scale bars represent 10 μm. Note the flat and highly condensed nucleus prior to hibernation and the initial phase of nucleus elimination in senescent queen. Abbreviations: cv, central vacuole; g, glycogen; ld, lipid droplet; m, mitochondria; n, nucleus; pg, protein granule; rer, rough endoplasmic reticulum.

The two principal cell types in the FB of *B*. *terrestris* queens are adipocytes and oenocytes (S1 Fig). Adipocytes are very large (for a comparison of adipocyte sizes among the studied stages, see S1 Table and S2 Fig) and more abundant compared to oenocytes. The two cell types are often intermingled, although some areas with predominance of a single cell type were observed. Oenocytes can be distinguished from adipocytes by the absence of both glycogen and lipid droplets and the presence of smooth endoplasmic reticulum and large amounts of irregular electron-dense granules. The ultrastructure of adipocytes in *B*. *terrestris* queens of various life stages is shown in Fig 1. The following description is based on the adipocytes of callow queens.

Each adipocyte is enclosed in a basement membrane formed by a single lamina about 70 nm thick. Basal invaginations are well-developed, up to 2 μm deep. Nuclei are highly irregular, formed by dispersed chromatin, and with only a few larger aggregates. The cytoplasm is rich in rough endoplasmic reticulum (RER) and free ribosomes; other secretory organelles were not observed. Mitochondria are abundant, scattered throughout the cytoplasm. Three kinds of inclusions were observed, with lipid droplets being the most abundant (up to 30 μm in diameter). Glycogen accumulations (in the form of moderately electron-dense rosettes) are located predominantly at the cell periphery. Proteins occur in two forms, as electron-dense granules and heavy electron-dense biocrystals, and both of these are relatively rare. Other observed structures comprise some microtubules and rarely lysosomes (small vacuoles of heterogeneous content). Each cell is equipped with a central vacuole [22] of variable size (between approximately 5 and 40 μm at the largest dimension), in which lipid droplets were infrequently observed to dissolve. Differences in FB structure among stages are summarized below.

Adipocytes in pharate queens reveal higher biosynthetic activity, as evidenced by more abundant RER, higher proportion of protein granules, and slightly larger nuclei filled with more dispersed chromatin with scarce aggregates. Also lysosomes are more abundant compared to callow queens.

Queens before hibernation possess the largest adipocytes of all stages (see S1 Table, S2 Fig), due to accumulation of enormous amounts of lipid droplets and glycogen. Nearly the whole cell volume is filled with these inclusions; lipid droplets become irregular due to their crowding. There is a low volume of cytoplasm, located only around highly condensed nucleus. Also the number of mitochondria decreases. The central vacuole is absent from this stage on.

Adipocytes in queens after hibernation are relatively small. The cells contain high amounts of glycogen, but the amount of lipid droplets is relatively low. Nuclei stay highly condensed, but the volume of the cytoplasm increases, as well as the number of mitochondria.

Adipocytes of egg-laying queens contain high amounts of RER and populous mitochondria. There is also a high amount of proteins (both, granules and biocrystals). Other inclusions comprise low amounts of glycogen and lipid droplets. Basal invaginations are very well-developed, often associated with mitochondria and reaching up to 10 μm deep. Nuclei are large, filled with dispersed chromatin and few larger aggregates.

Senescent queens reveal similar structure as observed in egg-laying queens, with exception of lower amounts of glycogen and lipid droplets, lower number of protein granules, and higher numbers of biocrystals. Rare observations suggest the possibility of adipocyte apoptosis, as few nuclei were observed containing electron dense vesicles representing probably lysosomes (Fig 1F).

**Fig 2:**
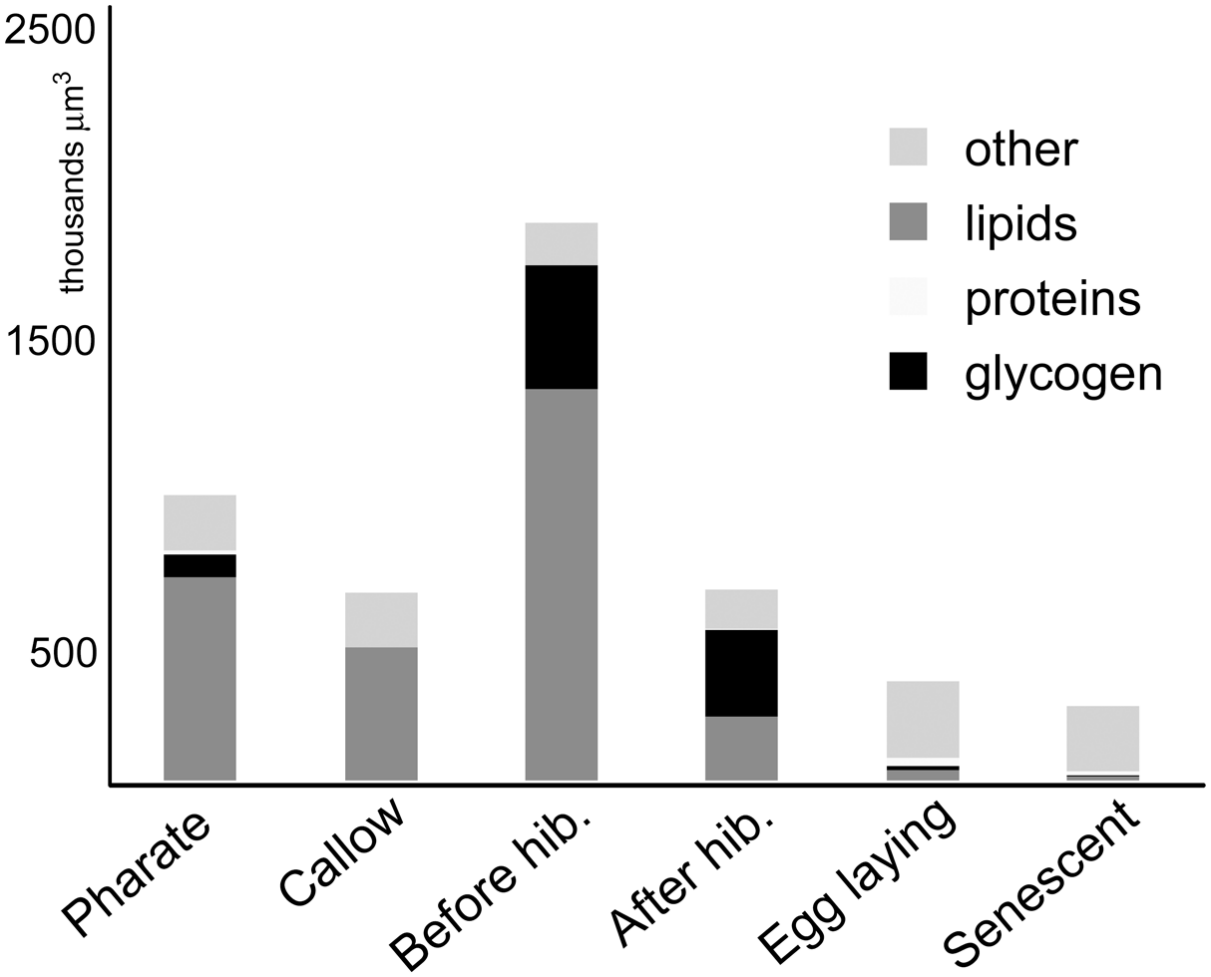
Comparison of cell volume and the proportions of glycogen, lipid droplets, and proteins at each life stage from transmission electron micrographs.

The individual life stages differ in the representation of storage inclusions (lipids, glycogen, and proteins). In most stages (pharate, callow, before and after hibernation), these inclusions formed almost the entire cell section (more than 90%). During the egg-laying and senescent stages, on the other hand, the FB contained just 10–20% of inclusions. The later life stages include well-developed machinery for protein production (voluminous RER), but they also contained a large volume of organelle-free cytoplasm. The results of semi-quantitative measurement of glycogen, TG (i.e., lipid droplets), and proteins (electron-dense granules) obtained from TEM are shown in Fig 2. Subsequent Monte Carlo testing of the CCA analyses of quantification data considered as unimodal (i.e., with gradient value higher than 1.5) revealed significant differences among particular age stages (*p* = 0.001) (S3 Fig). The greatest lipids content was found in queens before hibernation, pharate queens, and callow queens, while the largest amounts of glycogen were detected in queens before and after hibernation and in pharate queens (Fig 2). Surprisingly, the amount of glycogen is comparable in queens before and after overwintering. That is in contrast to a dramatic decrease in TG content (Fig 2). Egg-laying and senescent queens are the most similar stages and had the lowest contents of lipids and glycogen (S3 Fig). On the other hand, the content of proteins and other cell parts (cytoplasm, endoplasmic reticulum, etc.) was greatest in older queens (Fig 2).

Since only two individuals per stage were used for structural analyses, the significance of statistical results should not be overestimated, and these results serve as a comparison to chemical data.

### Trends in total lipids, TG, and body weights

**Fig 3:**
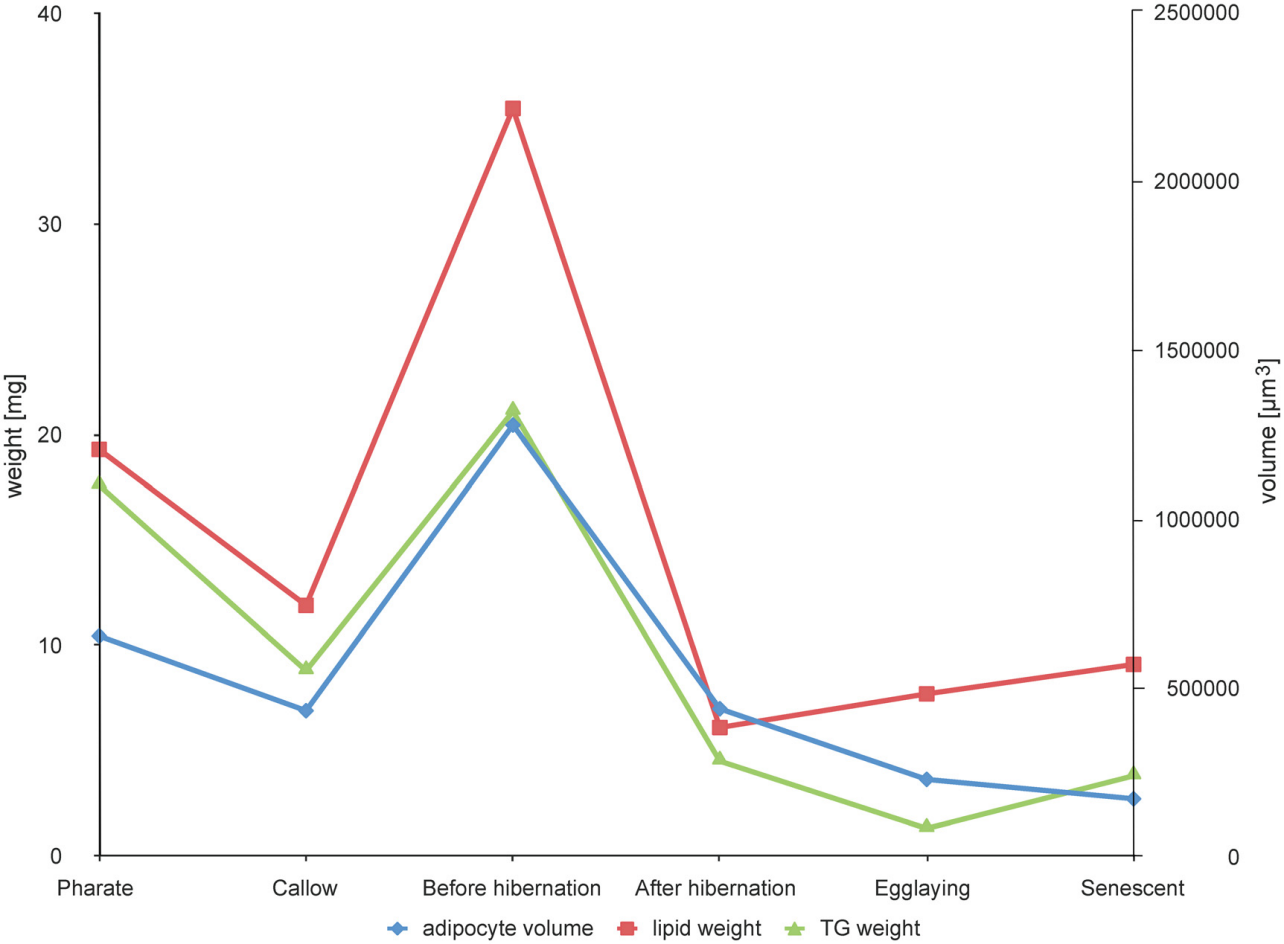
Trends of changes in adipocyte size, total lipid weight, and triacylglycerol (TG) weight in queens at different life stages (mean values, cf. Table 1 and S1 Table). Adipocyte volume in μm^3^; lipid weight in mg; TG weight in mg.

During the queen’s life, the greatest changes occur in the abdomen where the FB is located. The highest amount of total lipids was obtained from queens before hibernation (35.5 ± 17.0 mg), while the lowest amount (6.1 ± 4.1 mg) was recorded after hibernation (Table 1, Fig 3). After egg-laying and further aging, the amount of lipids again showed a slightly increasing trend. The HPLC/MS (APCI and ESI) analyses of all lipid classes (polar and nonpolar lipids) showed that reproductive stages contained higher proportions of polar lipids compared to others (S4 Fig). While TG were fairly abundant at all stages, PL and diacylglycerol content increased considerably in egg-laying and senescent queens (S4 Fig). The ratio between TG and PL varies among stages, the lowest value being observed in egg-laying queens. The body weight (without abdomen) changes only little during the queen lifespan with an insignificant maximum before hibernation (S2 Table).

**Table 1:** Total lipid and TG weights in the FB of queens at different life stages (mean ± SD).

	Total lipids	TG
Life stage	Weight [mg] (number of samples)	
Pharate	19.3 ± 5.2 (17)	17.6 ± 1.5 (5)
Callow	11.9 ± 2.7 (27)	8.8 ± 0.5 (6)
Before hibernation	35.5 ± 17.0 (19)	21.1 ± 3.9 (6)
After hibernation	6.1 ± 4.1 (19)	4.5 ± 3.9 (6)
Egg-laying	7.7 ± 2.1 (34)	1.3 ± 0.5 (6)
Senescent	9.1 ± 4.1 (16)	3.8 ± 3.7 (7)

TG, triacylglycerols

### TG analysis and fatty acid composition

**Fig 4:**
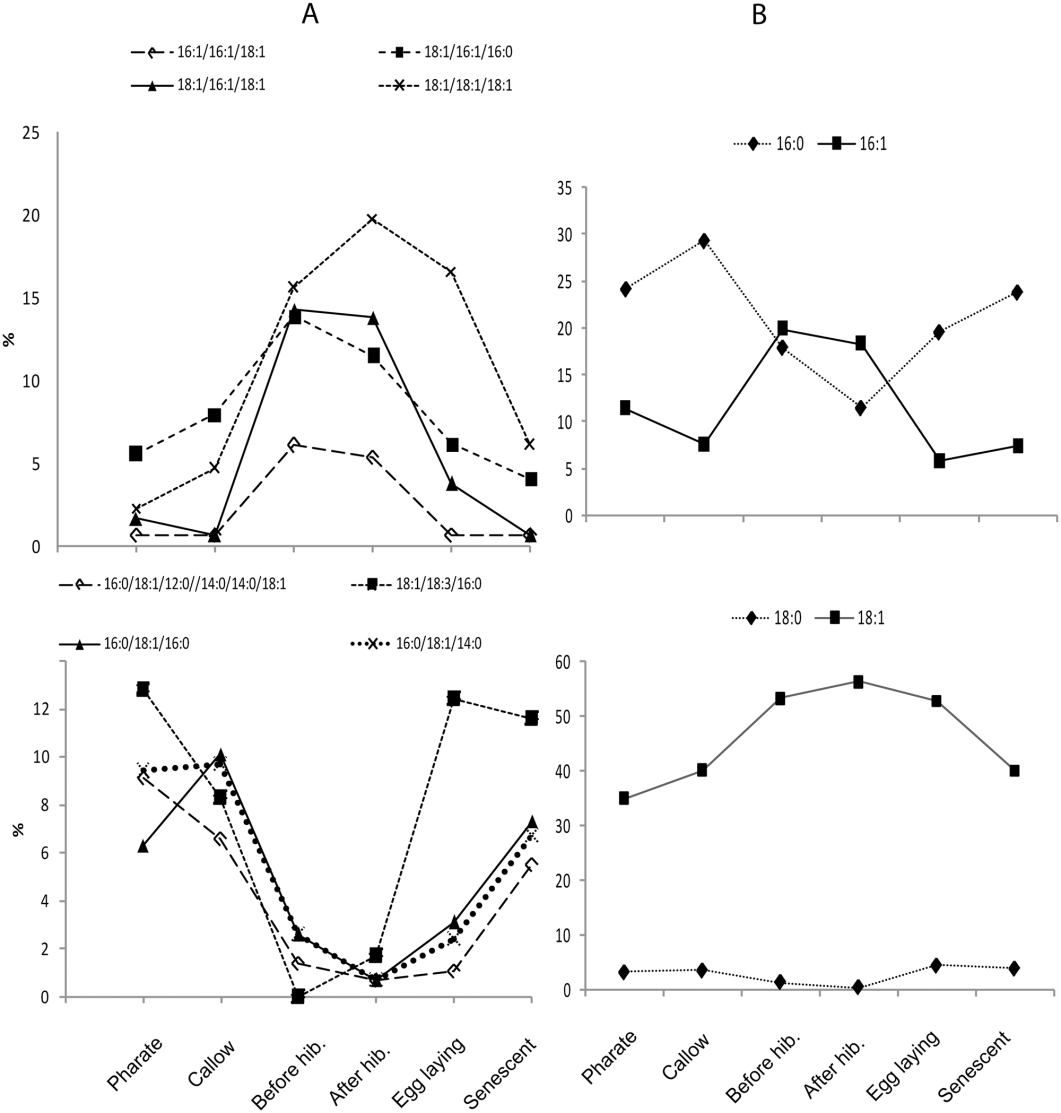
**(A) Trends in proportions of the most abundant triacylglycerols (TG).** Upper diagram shows the accumulation of particular TG in life stages involved in overwintering. Lower diagram shows a significant reduction in particular TG at the indicated life stages. **(B) Trends in proportions of saturated and monounsaturated fatty acids (FA) obtained by recalculation of HPLC/MS-APCI data.** Diagrams are focused on the most abundant FA, and some trends connected with overwintering are also observed. Generally, accumulation of monounsaturated FA and a decreased level of saturated FA in after- and before-hibernation life stages are obvious. Relative percentages were calculated from integrated peak areas of particular chromatographic peaks.

Altogether, 46 TG were identified and their equivalent carbon number (ECN) ranged between 40 and 52. The substances with lower ECN (40, 42) were present in smaller proportions (below 1%), while the most abundant TG were those of ECN 44 or 46 (S3 Table). The composition of TG blends differed both qualitatively and quantitatively among life stages (S5 Fig and S3, S4 Tables). Detailed study of the obtained dataset revealed that some of the most abundant TG showed stable relative proportions through the queens’ lifespan (S4 Table, group A). Several TG are obviously accumulated in relation to overwintering (especially those containing monounsaturated fatty acids; group B), while their saturated associates displayed the opposite trend (group C) (Fig 4A and S4 Table). Other components in the samples were present in amounts less than 1% or were not detectable at all at some stages (S3 Table). CCA statistical analysis of TG composition and the semi-quantification dataset followed by Monte Carlo testing revealed significant differences in particular life stages (*p* < 0.01).

The most abundant FA was oleic acid (18:1, 32–52%) followed by palmitic acid (16:0, 10–28%) and myristic acid (14:0, 2–12%; S5 Table). RDA statistics and subsequent Monte Carlo testing of the recalculated FA dataset did not show significant differences in the FA composition of FB at particular life stages. Particular FA did, however, display certain trends over the lifespan (Fig 4B). The proportion of saturated FA decreased in life stages connected to hibernation while the proportion of monounsaturated FA showed the opposite trend. The proportion of polyunsaturated FA (namely 18:3) did not change substantially during hibernation either in structural PL or in the storage TG (S5 Table and S6 Table). Across the lifespan, however, the proportions followed a pattern similar to that of saturated FA, reaching the lowest point before hibernation (S5 Table).

A detailed study examined membrane PL identification and quantification in relation to queen hibernation. We determined 6 species of lyso-PL, 18 species of phosphatidylethanolamines (PE), 13 species of phosphatidylcholines (PC), and 4 species of phosphatidylserines using HPLC/MS-ESI. The dominant lipid species was dioleoyl PC (PC 18:1/18:1), comprising almost 40% of all PL, followed by dioleoyl PE (PE 18:1/18:1) (at 15%). The result of the Monte Carlo testing revealed no significant differences between PL of queens before and during hibernation, and furthermore no trends were noted in our datasets (S6 Table).

## Discussion

Our results show that the FB physiology of bumblebee queens changes dramatically several times during their lifes. Both electron microscopy and chemical analyses of lipids highlighted seasonal variability in the amounts of stored lipid and showed those values reaching a maximum before hibernation. The results match the earlier work of Alford [23], who noted that queens possess the largest FB, that these increase in size as well as fat content after emergence, and that up to 80% of reserves are used during overwintering. There were significant differences among life stages in the content of basic inclusions. This is true especially for glycogen and TG observed in the form of lipid droplets [24].

The principal cells of the FB, the adipocytes (often called trophocytes, but the same term is also applied to the nurse cells of meroistic ovarioles), store large amounts of energy in the form of glycogen (a readily utilizable source of energy) or TG (a slowly accessible energy source but also the most dense in terms of energy-content [24]). The adipocytes also produce hexamerins (storage proteins), vitellogenins (glycolipoproteins, major components of yolk), and many other classes of proteins [25]. In other social Hymenoptera, the queen’s FB consists of up to 11 different FB cell types (*Monomorium* [26]; for review see Haunerland and Shirk [27]). Oenocytes are known to synthesize non-storage hydrocarbons [28]. They also reveal just a slight ultrastructural variation during the queen’s life, thus suggesting that they perform a similar function irrespective of age. While oenocytes occur in *B*. *terrestris* only in the peripheral FB, scarce adipocytes are present also near the gut and among the Malpighian tubules, and these differ considerably in their ultrastructure from peripheral adipocytes (JŠ, unpublished observation).

Protein content in adipocytes revealed a U-shaped development, with large amounts observed in the youngest and the oldest age categories. The dominant form of proteins in pharate queens (and to a lesser extent also in callow queens) consisted of electron-dense vesicles of heterogeneous content and less abundant biocrystals, probably linked with finalization in building basic body structures. In queens before and after hibernation, protein content is very low. On the other hand, large amounts of protein granules and biocrystals occur in reproductively active queens and, since the ultrastructural features of these inclusions are very similar to those of vitellogenins, which are proven to comprise part of the adipocytes in *Solenopsis* queens [26], we expect that these proteins form a substantial part of the developing egg yolk.

Over a queen’s lifetime, TG composition changes both quantitatively and qualitatively (see S5 Fig), and multivariate analysis of all detected TG (S3 Table) showed significant differences in TG profiles among particular life stages. Previous studies in insects have considered changes only in FA composition but not in intact lipids [29–31]. In bumblebee queens, the composition of intact TG molecules showed highly significant trends in association with overwintering.

Studies on FA composition of various insect tissues [32], as well as FA utilization during energy stress [33], development [24,32,34], and reproduction [33] indicate that particular FA indeed perform different roles in insect metabolism. Beenakkers et al. [35] reported on the existence of two different acylglycerol pools in FB with expected differences in the manner of their utilization. Metabolic TG belonging to group A (Fig 4) dominate also in the males' FB [15] while queen-specific TG change during the queens' life. This may indicate their relationship to overwintering and possibly also to future egg formation and/or development with their special metabolic requirements beyond the needs of short-lived males.

The most significant changes in lipid composition are undoubtedly connected to hibernation (Fig 4A). The fat reserves are clearly of great importance for insects in order to meet their energy demands during hibernation [36]. Naturally occurring queens could be expected to deplete even more energy reserves during diapause as hibernation lasts longer in nature (ca 8 weeks compared to 5 weeks in laboratory conditions). The amount of fat used during hibernation also depends on overwintering temperature. Vesterlund et al. [37] found that at higher winter temperatures metabolism (in terms of enzyme activity) is faster and more fat is therefore metabolized compared to cold winters. As for FA composition, we could see clear trends in qualitative changes: Monounsaturated FA level increased before hibernation at the expense of saturated analogues that are preferably consumed during winter (Fig 4B). Therefore, the utilization of FA for energy demands during hibernation is nonrandom and involves lipases with selective preferences. A similar phenomenon has been described in *Locusta migratoria* showing selective mobilization of FA by adipokinetic hormones [33].

Substantial amounts of glycogen are present in three of queens’ life stages: pharate, before hibernation, and after hibernation. Pharate queens deplete glycogen storage for metamorphosis such that only traces remain in freshly emerged queens’ FB. After emergence and during their sexually active life phase, queens build up glycogen again (Fig 2). The level of glycogen surprisingly does not drop during hibernation, indicating that hibernation expenses are covered exclusively by TG. Glycogen stores may also be used as a source of polyol cryoprotectants [18,37].

The common response of an insect to low temperatures consists in membrane restructuring [16,19] and/or the accumulation of polyols [17,18]. Inasmuch as our HPLC/MS–ESI data on PL membrane composition in overwintering queens revealed no significant changes, carbohydrate-based cryoprotectant accumulation is expected to take place in overwintering processes. Low temperatures may promote the conversion of glycogen stores into glycerol [38], sorbitol, or ribitol [18]. On the contrary, the gall fly *Eurosta solidaginis* is known to reconvert carbohydrate-based cryoprotectants back into glycogen at the end of the cold season [39]. Similar mechanisms should not therefore be excluded in overwintering queens, and this may explain the high levels of glycogen before and after hibernation.

A combination of optical and transmission electron microscopy with analytical chemistry techniques provides detailed insight into the structural and functional changes of *B*. *terrestris* queens’ FB during adulthood. In relation to overwintering and reproduction, bumblebee queens undergo substantial physiological changes which are accompanied by changes in lipid amounts and composition as well as changes in glycogen content. The most pronounced changes and trends in cell structure as well as lipid composition are definitely connected to hibernation, the stage imposing the highest energy demands during the queen’s life.

## Supporting Information

S1 Dataset Raw data for each sample (PL analytical data, TG analytical data, FA recalculations, adipocyte size measurements and calculations, weights of total lipids, TG, and bodies without abdomens). (XLSX)

S1 Fig Optical microscopy of the fat body of *B*. *terrestris* queens: pharate (A), callow (B), before hibernation (C), after hibernation (D), egg-laying (E), and senescent (F). Scale bar represents 200 μm. Abbreviations: a, adipocyte; c, cuticle; e, oenocyte; m, muscle; t, trachea.
(PDF)

S2 Fig Comparison of whole adipocytes volumes in particular life stages of *B*. *terrestris* queens. Data represent means ± S.D. (n = 18) of whole cells volumes. Significantly different expression levels are indicated by different letters. The data passed the Kolmogorov-Smirnov test of normality and subsequently were subjected to One-way ANOVA with Tukey's multiple comparison test (P < 0.0001).
(PDF)

S3 Fig Generated data visualization by CCA analysis shows the relationship between species (empty triangles) and samples (solid triangles). (PDF)

S4 Fig Base peak chromatograms of total lipid extracts of fat bodies obtained from *B*. *terrestris* queens: pharate, callow, before hibernation, after hibernation, egg-laying, and senescent. Bidirectional arrows indicate the expansion of each type of lipid in chromatogram. Abbreviations: DG–diacylglycerols; LysoPL–lysophospholipids; PC–phosphatidylcholines; PE–phosphatidylethanolamines; PL–phospholipids; PS–phosphatidylserines; TG–triacylglycerols.
(PDF)

S5 Fig Base peak chromatograms of TG isolated from fat body of *B*. *terrestris* queens in various life stages: (A) pharate queen, (B) callow queen, (C) queen before hibernation, (D) queen after hibernation, (E) egg-laying queen, (F) senescent queen. M–myristic acid, 14:0; P–palmitic acid, 16:0; Po–palmitoleic acid, 16:1; S–stearic acid, 18:0; O–oleic acid, 18:1; Ln–linolenic acid, 18:3.
(PDF)

S1 Table Volumes of whole FB cells and representation of cytoplasm and inclusions in particular life stages of *B*. *terrestris* queens. In each stage, 2 queens were selected and size of 9 adipocytes was measured in each.
(PDF)

S2 Table Body weight (without abdomen) of queens in different life stages (mean values ± standard deviation). (PDF)

S3 Table Relative composition of triacylglycerols in *Bombus terrestris* queen fat body in different life phases identified using reversed-phase HPLC/MS-APCI (n = 10). ECN = equivalent carbon number, CN = total number of carbon atoms, DB = total number of double bonds.
(PDF)

S4 Table List of most abundant TG in *B*. *terrestris* queens’ fat bodies showing some proportional trends during the lifespan. Group A is relatively stable, while groups B and C correlated with hibernation period of life (i.e., accumulation for B and consumption for C).
(PDF)

S5 Table List of fatty acid (FA) composition of intact TG molecules and recalculated ratio of particular FA in fat bodies of *B*. *terrestris* queens in different life phases (relative %). (PDF)

S6 Table Phospholipid membrane composition of fat body of *B*. *terrestris* queens before and during hibernation obtained by HPLC/MS-ESI technique (relative % in phospholipid fraction; n = 5). PE, phosphatidylethanolamine; PC, phosphatidylcholine; PS, phosphatidylserine.
(PDF)
