# Supplementary material for: Seasonal Dynamics in the Chemistry and Structure of the Fat Bodies of Bumblebee Queens
Source: PLoS One. 2015 Nov 11;10(11):e0142261. doi: 10.1371/journal.pone.0142261 (PMC4641598; doi:10.1371/journal.pone.0142261)
Supplement: S1 Fig — Optical microscopy of the fat body of B. terrestris queens: pharate (A), callow (B), before hibernation (C), after hibernation (D), egg-laying (E), and senescent (F). Scale bar represents 200 μm. Abbreviations: a, adipocyte; c, cuticle; e, oenocyte; m, muscle; t, trachea. (PDF) [file pone.0142261.s002.pdf]

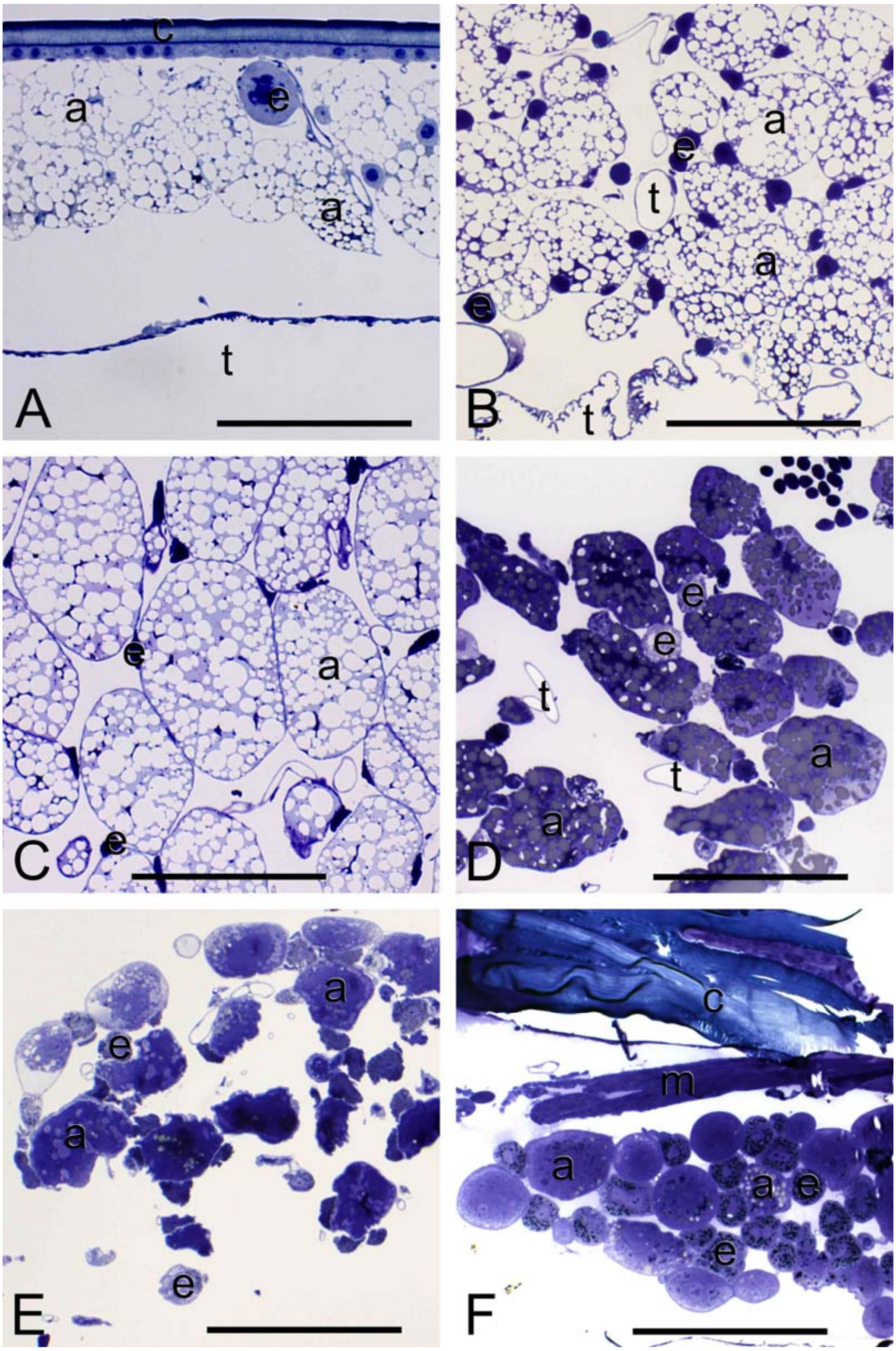

S1 Fig. Optical microscopy of the fat body of *B. terrestris* queens, pharate (A), callow (B), before hibernation (C), after hibernation (D), egg-laying (E) and senescent (F). Scale bar represents 200 µm. Abbreviations: a, adipocyte; c, cuticle; e, oenocyte; m, muscle; t, trachea.
