# Supplementary material for: Seasonal Dynamics in the Chemistry and Structure of the Fat Bodies of Bumblebee Queens
Source: PLoS One. 2015 Nov 11;10(11):e0142261. doi: 10.1371/journal.pone.0142261 (PMC4641598; doi:10.1371/journal.pone.0142261)
Supplement: S2 Fig — Data represent means ± S.D. (n = 18) of whole cells volumes. Significantly different expression levels are indicated by different letters. The data passed the Kolmogorov-Smirnov test of normality and subsequently were subjected to One-way ANOVA with Tukey's multiple comparison test (P < 0.0001). (PDF) [file pone.0142261.s003.pdf]

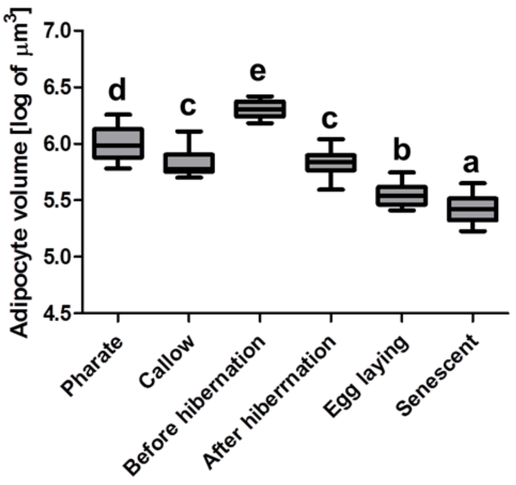

S2 Fig. Comparison of the whole adipocytes volumes in particular life stages of *B. terrestris* queens. Whiskers represent the minimum and maximum values of whole cells volumes (n=18). Significantly different groups are indicated by different letters. Log-transformed data were subjected to One-way ANOVA with Tukey's post-hoc multiple comparison test (P < 0.0001).
