# Supplementary material for: Seasonal Dynamics in the Chemistry and Structure of the Fat Bodies of Bumblebee Queens
Source: PLoS One. 2015 Nov 11;10(11):e0142261. doi: 10.1371/journal.pone.0142261 (PMC4641598; doi:10.1371/journal.pone.0142261)
Supplement: S3 Fig — (PDF) [file pone.0142261.s004.pdf]

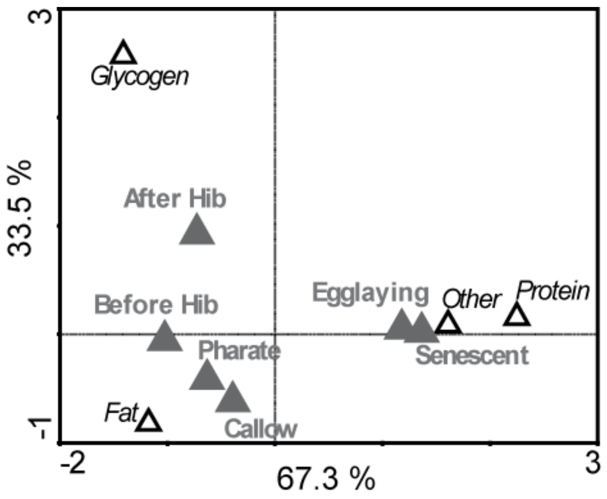

S3 Fig. Generated data visualization by CCA analysis shows the relationship between species (empty triangels) and samples (solid triangles).
