# Supplementary material for: Seasonal Dynamics in the Chemistry and Structure of the Fat Bodies of Bumblebee Queens
Source: PLoS One. 2015 Nov 11;10(11):e0142261. doi: 10.1371/journal.pone.0142261 (PMC4641598; doi:10.1371/journal.pone.0142261)
Supplement: S4 Fig — Bidirectional arrows indicate the expansion of each type of lipid in chromatogram. Abbreviations: DG–diacylglycerols; LysoPL–lysophospholipids; PC–phosphatidylcholines; PE–phosphatidylethanolamines; PL–phospholipids; PS–phosphatidylserines; TG–triacylglycerols. (PDF) [file pone.0142261.s005.pdf]

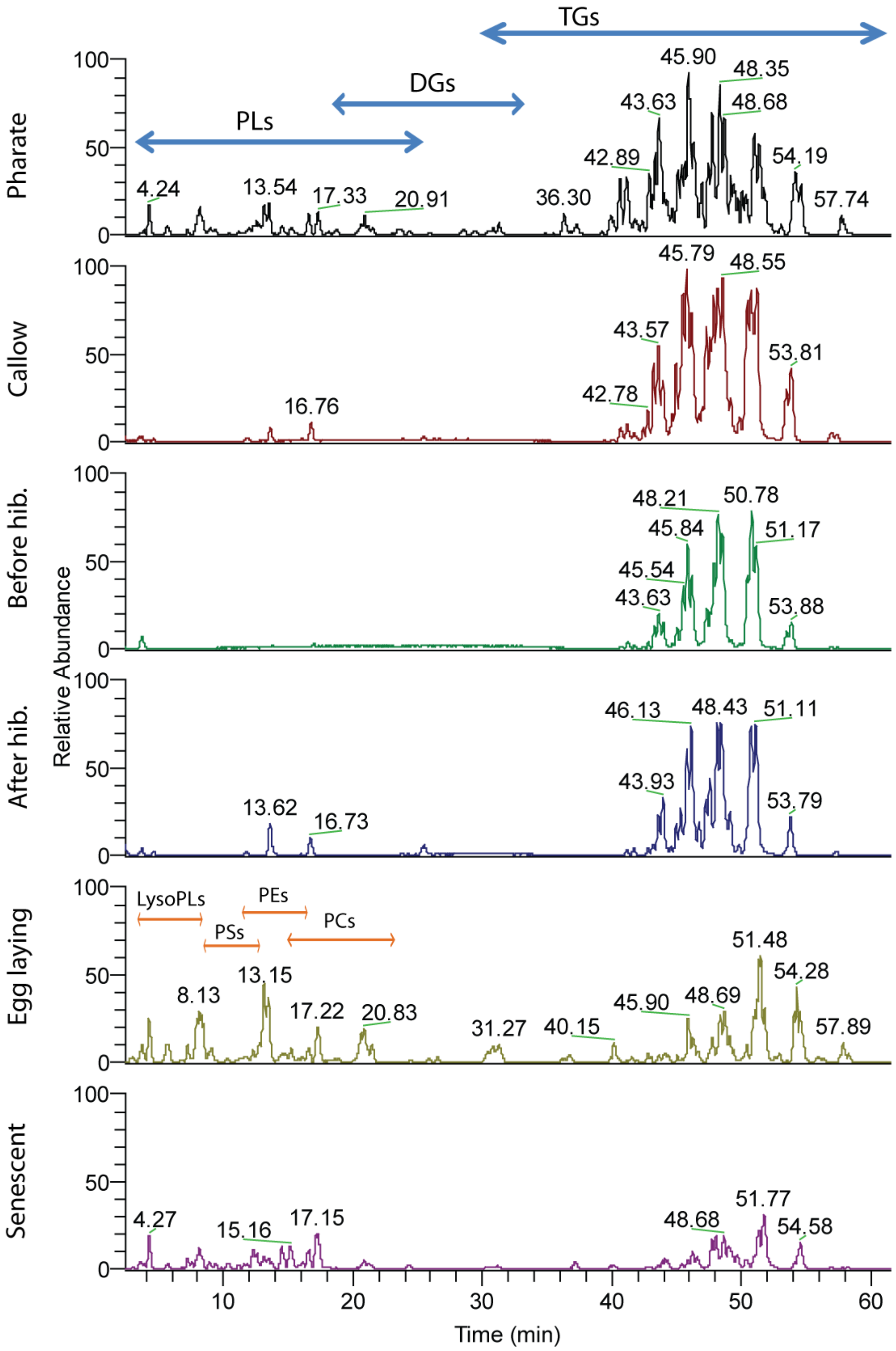

S4 Fig. Base peak chromatograms of total lipid extracts of fat bodies obtained from *B. terrestris* queens: pharate, callow, before hibernation, after hibernation, egg-laying, and senescent ones. Bidirectional arrows indicate the expansion of each type of lipid in chromatogram. Abbreviations: DG - diacylglycerols; LysoPL - lysophospholipides; PC – phospahtidycholines PE - phosphatidylethanolamines; PL - phospholipids; PS - phosphatidylserines; TG- triacylglycerols.
