# Supplementary material for: Seasonal Dynamics in the Chemistry and Structure of the Fat Bodies of Bumblebee Queens
Source: PLoS One. 2015 Nov 11;10(11):e0142261. doi: 10.1371/journal.pone.0142261 (PMC4641598; doi:10.1371/journal.pone.0142261)
Supplement: S5 Fig — Base peak chromatograms of TG isolated from fat body of B. terrestris queens in various life stages: (A) pharate queen, (B) callow queen, (C) queen before hibernation, (D) queen after hibernation, (E) egg-laying queen, (F) senescent queen. M–myristic acid, 14:0; P–palmitic acid, 16:0; Po–palmitoleic acid, 16:1; S–stearic acid, 18:0; O–oleic acid, 18:1; Ln–linolenic acid, 18:3. (PDF) [file pone.0142261.s006.pdf]

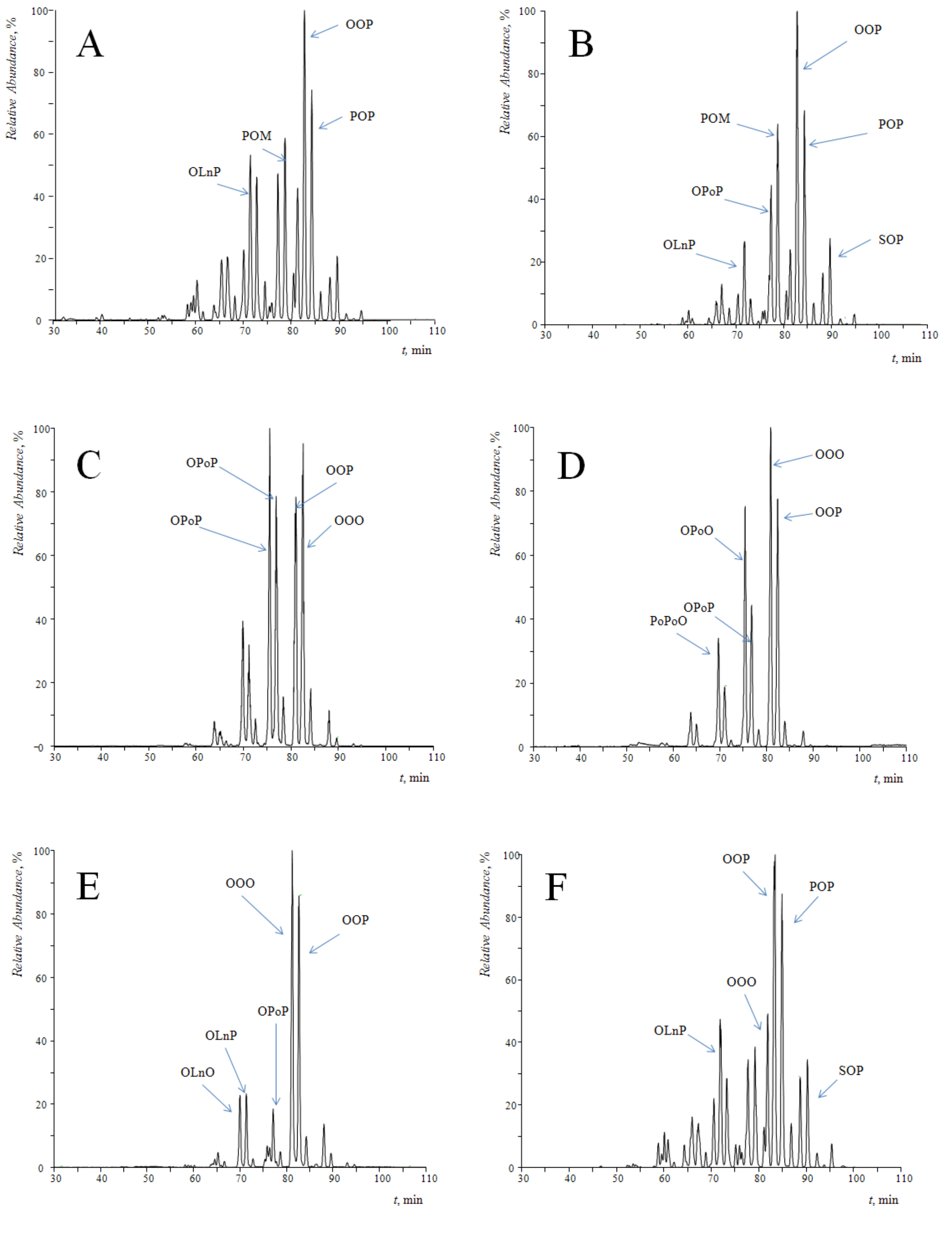

S5 Fig. Base peak chromatograms of TG isolated from fat body of *B. terrestris* queens in various life stages: (A) pharate queen, (B) callow queen, (C) queen before hibernation, (D) queen after hibernation, (E) egg-laying queen, (F) senescent queen. M – myristic acid, 14:0; P – palmitic acid, 16:0; Po – palmitoleic acid, 16:1; S – stearic acid, 18:0; O – oleic acid, 18:1; Ln – linolenic acid, 18:3.
