# Supplementary material for: Seasonal Dynamics in the Chemistry and Structure of the Fat Bodies of Bumblebee Queens
Source: PLoS One. 2015 Nov 11;10(11):e0142261. doi: 10.1371/journal.pone.0142261 (PMC4641598; doi:10.1371/journal.pone.0142261)
Supplement: S1 Table — In each stage, 2 queens were selected and size of 9 adipocytes was measured in each. (PDF) [file pone.0142261.s007.pdf]

S1 Table. Volumes of whole cell, inclusion, and droplets in particular life stages of *B. terrestris* queens. In each stage, 2 queens were selected, size of 9 adipocytes measured in each of them.

	Lipid [μm^3]	Glycogen [μm^3]	Protein [μm^3]	Other [μm^3]	Whole cell [μm^3]
Pharate	744785	86222	13264	200828	1045099
Callow	487524	912	625	200700	689761
Before hibernation	1437412	454054	476	153366	2045309
After hibernation	235588	318880	1350	143471	699290
Egglaying	37711	14357	30336	280702	363106
Senescent	15140	3074	13622	238977	270813
