# Supplementary material for: Seasonal Dynamics in the Chemistry and Structure of the Fat Bodies of Bumblebee Queens
Source: PLoS One. 2015 Nov 11;10(11):e0142261. doi: 10.1371/journal.pone.0142261 (PMC4641598; doi:10.1371/journal.pone.0142261)
Supplement: S2 Table — (PDF) [file pone.0142261.s008.pdf]

S2 Table. Body weight (without abdomen) of queens in different life stages (mean values ± standard deviation).

Life stage	Body weight [mg] (number of samples)
Pharate	95.3 ± 4.5 (17)
Callow	90.1 ± 4.7 (27)
Before hibernation	118.5 ± 10.1 (19)
After hibernation	92.2 ± 7.2 (19)
Egg-laying	109.4 ± 8.8 (34)
Senescent	112.2 ± 8.3 (16)
