# Supplementary material for: Seasonal Dynamics in the Chemistry and Structure of the Fat Bodies of Bumblebee Queens
Source: PLoS One. 2015 Nov 11;10(11):e0142261. doi: 10.1371/journal.pone.0142261 (PMC4641598; doi:10.1371/journal.pone.0142261)
Supplement: S3 Table — ECN = equivalent carbon number, CN = total number of carbon atoms, DB = total number of double bonds. (PDF) [file pone.0142261.s009.pdf]

**S3 Table.** Relative composition of triacylglycerols in *Bombus terrestris* queen fat body in different life phases identified using reversed-phase HPLC/MS-APCI (n = 10). ECN = equivalent carbon number, CN = total number of carbon atoms, DB = total number of double bonds.

| | | Queen's life phase | Pharate | | Callow | | Before hibernation | | After hibernation | | Egg-laying | | Senescent | |
|---|---|---|---|---|---|---|---|---|---|---|---|---|---|---|
| ECN | CN:DB | TG | Mean | sd | Mean | sd | Mean | sd | Mean | sd | Mean | sd | Mean | sd |
| 38 | 40:1 | 16:1/ 12:0/ 12:0 | 0.00 | 0.00 | 0.00 | 0.00 | 0.00 | 0.00 | 0.00 | 0.00 | 0.08 | 0.10 | 0.00 | 0.00 |
| 38 | 44:3 | 18:2/12:1/14:0 | 0.38 | 0.08 | 0.00 | 0.00 | 0.00 | 0.00 | 0.00 | 0.00 | 0.04 | 0.04 | 0.11 | 0.13 |
| 38 | 48:5 | 18:3/ 16:1/ 14:1 | 0.00 | 0.00 | 0.00 | 0.00 | 0.00 | 0.00 | 0.00 | 0.00 | 0.01 | 0.02 | 0.00 | 0.00 |
| 38 | 50:6 | 18:3/18:3/14:0 | 0.25 | 0.07 | 0.00 | 0.00 | 0.00 | 0.00 | 0.00 | 0.00 | 0.03 | 0.02 | 0.09 | 0.11 |
| 40 | 40:0 | 12:0/16:0/12:0//14:0/14:0/12:0 | 0.13 | 0.05 | 0.09 | 0.06 | 0.00 | 0.00 | 0.00 | 0.00 | 0.09 | 0.12 | 0.08 | 0.13 |
| 40 | 42:1 | 14:1/16:0/12:0//14:0/14:0/14:1 | 0.24 | 0.09 | 0.25 | 0.07 | 0.00 | 0.00 | 0.04 | 0.02 | 0.05 | 0.08 | 0.00 | 0.00 |
| 40 | 44:2 | 18:2/12:0/14:0 | 0.00 | 0.00 | 0.00 | 0.00 | 0.10 | 0.07 | 0.13 | 0.04 | 0.00 | 0.00 | 0.00 | 0.00 |
| 40 | 56:3 | 14:0/18:3/14:0//16:0/18:3/12:0// 18:3/12:0/16:0 | 1.97 | 0.53 | 0.60 | 0.15 | 0.18 | 0.18 | 0.11 | 0.06 | 0.27 | 0.23 | 1.02 | 0.82 |
| 40 | 50:5 | 18:2/18:2/14:1 | 0.37 | 0.13 | 0.00 | 0.00 | 0.00 | 0.00 | 0.00 | 0.00 | 0.03 | 0.05 | 0.00 | 0.00 |
| 40 | 50:5 | 20:3/14:1/16:1 | 0.00 | 0.00 | 0.00 | 0.00 | 0.29 | 0.13 | 0.26 | 0.06 | 0.00 | 0.00 | 0.42 | 0.37 |
| 40 | 52:4 | 18:2/18:2/16:0 | 1.43 | 0.44 | 0.42 | 0.08 | 0.16 | 0.07 | 0.24 | 0.09 | 0.33 | 0.21 | 0.45 | 0.44 |
| 40 | 52:6 | 18:2/18:3/ 16:1 | 1.19 | 0.44 | 0.89 | 0.45 | 0.11 | 0.04 | 0.07 | 0.01 | 0.20 | 0.14 | 0.76 | 0.82 |
| 42 | 46:2 | 16:1/14:0/16:1 | 0.26 | 0.08 | 0.36 | 0.02 | 1.05 | 0.62 | 1.17 | 0.37 | 0.00 | 0.00 | 0.38 | 0.55 |
| 42 | 42:0 | 14:0/16:0/12:0//14:0/14:0/14:0 | 0.32 | 0.14 | 0.59 | 0.15 | 0.00 | 0.00 | 0.01 | 0.00 | 0.10 | 0.15 | 0.59 | 0.78 |
| 42 | 44:1 | 14:0/16:1/14:0 | 1.56 | 1.25 | 1.95 | 0.32 | 0.07 | 0.05 | 0.36 | 0.13 | 0.50 | 0.61 | 1.89 | 1.81 |
| 42 | 48:3 | 16:1/16:1/16:1 | 3.46 | 0.75 | 0.35 | 0.45 | 1.74 | 1.07 | 1.16 | 0.30 | 0.07 | 0.06 | 1.77 | 1.59 |
| 42 | 48:3 | 18:2, 16:1, 14:0 | 4.48 | 0.74 | 1.12 | 0.65 | 0.00 | 0.00 | 0.15 | 0.04 | 0.72 | 0.27 | 0.78 | 1.04 |
| 42 | 50:4 | 18:1/16:1/16:2 | 4.69 | 1.11 | 1.85 | 0.36 | 0.39 | 0.16 | 0.50 | 0.56 | 1.84 | 0.98 | 2.67 | 1.48 |
| 42 | 50:4 | 18:3/18:1/14:0 | 0.00 | 0.00 | 0.04 | 0.02 | 0.92 | 0.31 | 0.72 | 0.59 | 0.00 | 0.00 | 0.06 | 0.11 |
| 42 | 52:5 | 18:2/ 18:2/ 16:1 | 0.86 | 0.38 | 0.26 | 0.08 | 1.67 | 0.51 | 1.42 | 0.15 | 1.16 | 0.90 | 0.52 | 0.29 |
| 42 | 52:5 | 18:3/18:2/16:0 | 0.91 | 0.47 | 0.64 | 0.23 | 0.37 | 0.12 | 0.16 | 0.04 | 0.00 | 0.00 | 1.12 | 0.65 |
| 42 | 54:6 | 18:2/18:2/18:2 | 0.62 | 0.47 | 0.37 | 0.19 | 0.99 | 0.23 | 0.30 | 0.05 | 0.39 | 0.25 | 0.81 | 0.51 |
| 42 | 54:6 | 18:2/18:3/ 18:1 | 0.00 | 0.00 | 0.09 | 0.11 | 0.00 | 0.00 | 0.00 | 0.00 | 0.00 | 0.00 | 0.00 | 0.00 |

| | | | | | | | | | | | | | | |
|---|---|---|---|---|---|---|---|---|---|---|---|---|---|---|
| 44 | 44:0 | 16:0/12:0/16:0//14:0/14:0/16:0 | 0.56 | 0.23 | 1.08 | 0.17 | 0.00 | 0.00 | 0.03 | 0.02 | 0.20 | 0.26 | 0.91 | 1.12 |
| 44 | 46:1 | 16:0/18:1/12:0//14:0/14:0/18:1 | 9.10 | 0.20 | 5.68 | 2.44 | 0.27 | 0.18 | 1.43 | 0.43 | 2.10 | 1.79 | 5.54 | 4.21 |
| 44 | 48:2 | 18:1/14:0/16:1//16:1/16:1/16:0 | 2.55 | 0.42 | 2.34 | 1.15 | 4.95 | 1.96 | 5.62 | 1.00 | 1.49 | 0.13 | 2.08 | 1.26 |
| 44 | 50:3 | 16:1/16:1/18:1 | 0.54 | 0.14 | 0.56 | 0.27 | 5.92 | 2.00 | 6.02 | 0.89 | 1.28 | 0.14 | 0.89 | 0.52 |
| 44 | 50:3 | 18:1/18:1/14:1 | 5.06 | 0.49 | 1.64 | 0.75 | 1.07 | 0.39 | 0.00 | 0.00 | 0.00 | 0.00 | 2.90 | 1.28 |
| 44 | 52:4 | 18:1/18:3/16:0 | 12.63 | 1.71 | 8.08 | 1.96 | 1.72 | 0.26 | 3.35 | 0.87 | 12.14 | 2.41 | 11.56 | 4.12 |
| 44 | 52:4 | 18:1/18:2/16:1 | 0.00 | 0.00 | 0.00 | 0.00 | 2.64 | 0.69 | 0.41 | 0.08 | 0.00 | 0.00 | 0.00 | 0.00 |
| 44 | 54:5 | 18:1/18:3/18:1 | 4.96 | 0.96 | 2.98 | 0.37 | 5.90 | 1.81 | 4.01 | 0.39 | 9.71 | 2.77 | 5.54 | 3.21 |
| 44 | 54:5 | 18:2/ 18:3/ 18:0 | 0.00 | 0.00 | 0.17 | 0.04 | 0.00 | 0.00 | 0.00 | 0.00 | 0.00 | 0.00 | 0.00 | 0.00 |
| 46 | 46:0 | 16:0/14:0/16:0 | 0.82 | 0.19 | 1.47 | 0.28 | 0.00 | 0.00 | 0.05 | 0.01 | 0.43 | 0.63 | 0.85 | 0.83 |
| 46 | 48:1 | 16:0/18:1/14:0 | 9.49 | 0.80 | 9.84 | 1.32 | 0.43 | 0.24 | 2.61 | 0.36 | 3.17 | 1.52 | 6.71 | 2.48 |
| 46 | 50:2 | 18:1/16:1/16:0 | 5.39 | 0.69 | 7.84 | 0.74 | 11.84 | 2.82 | 14.02 | 0.46 | 5.99 | 1.29 | 4.09 | 4.09 |
| 46 | 50:2 | 16:0/16:0/18:2 | 0.00 | 0.00 | 0.35 | 0.10 | 0.00 | 0.00 | 0.00 | 0.00 | 0.00 | 0.00 | 0.00 | 0.00 |
| 46 | 52:3 | 18:1/16:1/18:1 | 1.38 | 0.74 | 1.07 | 0.38 | 14.66 | 2.14 | 13.22 | 2.29 | 3.97 | 1.06 | 1.45 | 1.77 |
| 46 | 52:3 | 18:2/ 18:1/ 16:0 | 0.00 | 0.00 | 2.07 | 0.45 | 2.40 | 0.56 | 1.02 | 0.22 | 1.22 | 0.32 | 2.70 | 1.02 |
| 46 | 54:4 | 18:1/18:1/18:2 | 0.49 | 0.14 | 0.74 | 0.15 | 4.54 | 1.01 | 1.04 | 0.08 | 1.56 | 0.95 | 1.25 | 0.84 |
| 46 | 54:4 | 18:2/18:0/18:2 | 2.16 | 0.71 | 1.68 | 0.39 | 0.33 | 0.09 | 0.00 | 0.00 | 4.61 | 1.71 | 2.44 | 1.37 |
| 48 | 48:0 | 16:0/16:0/16:0 | 0.32 | 0.17 | 1.01 | 0.41 | 0.00 | 0.00 | 0.34 | 0.16 | 0.26 | 0.30 | 0.71 | 0.57 |
| 48 | 50:1 | 16:0/18:1/16:0 | 5.68 | 2.17 | 10.40 | 2.06 | 0.35 | 0.17 | 2.62 | 0.32 | 2.91 | 0.59 | 7.05 | 2.03 |
| 48 | 52:2 | 18:1/18:1/16:0 | 8.05 | 2.48 | 17.86 | 2.29 | 13.54 | 1.35 | 17.55 | 1.30 | 17.50 | 5.75 | 15.03 | 5.34 |
| 48 | 54:3 | 18:1/18:1/18:1 | 2.17 | 0.29 | 4.72 | 0.33 | 19.52 | 6.38 | 15.47 | 1.52 | 16.13 | 2.67 | 6.09 | 3.98 |
| 50 | 50:0 | 16:0/18:0/16:0 | 0.90 | 0.28 | 0.66 | 0.65 | 0.00 | 0.00 | 0.42 | 0.05 | 0.00 | 0.00 | 0.37 | 0.23 |
| 50 | 52:1 | 18:0/18:1/16:0 | 3.02 | 1.25 | 4.47 | 0.54 | 0.00 | 0.00 | 0.88 | 1.17 | 3.06 | 1.97 | 4.19 | 1.96 |
| 50 | 54:2 | 18:1/18:1/18:0 | 1.26 | 0.43 | 2.78 | 0.56 | 1.22 | 0.40 | 1.67 | 0.50 | 5.26 | 1.74 | 3.39 | 1.46 |
| 50 | 56:3 | 18:1/20:1/18:1 | 0.00 | 0.00 | 0.00 | 0.00 | 0.66 | 0.31 | 0.19 | 0.03 | 0.00 | 0.00 | 0.00 | 0.00 |
| 52 | 54:1 | 18:0/18:0/18:1 | 0.35 | 0.10 | 0.59 | 0.10 | 0.00 | 0.00 | 0.06 | 0.01 | 1.09 | 0.46 | 0.76 | 0.41 |

sd = standard deviation
