# Supplementary material for: Seasonal Dynamics in the Chemistry and Structure of the Fat Bodies of Bumblebee Queens
Source: PLoS One. 2015 Nov 11;10(11):e0142261. doi: 10.1371/journal.pone.0142261 (PMC4641598; doi:10.1371/journal.pone.0142261)
Supplement: S4 Table — Group A is relatively stable, while groups B and C correlated with hibernation period of life (i.e., accumulation for B and consumption for C). (PDF) [file pone.0142261.s010.pdf]

S4 Table. List of most abundant TGs in *B. terrestris* queens' fat bodies showing some proportional trends during the lifespan. Group A is relatively stable, while groups B and C correlated with hibernation period of life (i.e., accumulation for B and consumption for C).

			Queen's life phase (relative % in TG fraction)											
			Pharate		Callow		Before hibernation		After hibernation		Egg-laying		Senescent	
CN:DB / Triacylglycerols			Mean	sd	Mean	sd	Mean	sd	Mean	sd	Mean	sd	Mean	sd
Group A	48:2	18:1/14:0/16:1//16:1/16:1/16:0	2.6	0.42	2.3	1.15	4.9	1.96	5.6	1.00	1.5	0.13	2.1	1.26
	54:5	18:1/18:3/18:1	5.0	0.96	3.0	0.37	5.9	1.81	4.0	0.39	9.7	2.77	5.5	3.21
	52:2	18:1/18:1/16:0	8.1	2.48	17.9	2.29	13.5	1.35	17.6	1.30	17.5	5.75	15.0	5.34
Group B	50:3	16:1/16:1/18:1	0.5	0.14	0.6	0.27	5.9	2.00	6.0	0.89	1.3	0.14	0.9	0.52
	50:2	18:1/16:1/16:0	5.4	0.69	7.8	0.74	11.8	2.82	14.0	0.46	6.0	1.29	4.1	4.09
	52:3	18:1/16:1/18:1	1.4	0.74	1.1	0.38	14.7	2.14	13.2	2.29	4.0	1.06	1.4	1.77
	54:3	18:1/18:1/18:1	2.2	0.29	4.7	0.33	19.5	6.38	15.5	1.52	16.1	2.67	6.1	3.98
Group C	46:1	16:0/18:1/12:0//14:0/14:0/18:1	9.1	0.20	5.7	2.44	0.3	0.18	1.4	0.43	2.1	1.79	5.5	4.21
	52:4	18:1/18:3/16:0	12.6	1.71	8.1	1.96	1.7	0.26	3.3	0.87	12.1	2.41	11.6	4.12
	48:1	16:0/18:1/14:0	9.5	0.80	9.8	1.32	0.4	0.24	2.6	0.36	3.2	1.52	6.7	2.48
	50:1	16:0/18:1/16:0	5.7	2.17	10.4	2.06	0.4	0.17	2.6	0.32	2.9	0.59	7.1	2.03

sd = standard deviation
