# Supplementary material for: Seasonal Dynamics in the Chemistry and Structure of the Fat Bodies of Bumblebee Queens
Source: PLoS One. 2015 Nov 11;10(11):e0142261. doi: 10.1371/journal.pone.0142261 (PMC4641598; doi:10.1371/journal.pone.0142261)
Supplement: S5 Table — (PDF) [file pone.0142261.s011.pdf]

S5 Table. List of fatty acid (FA) composition of intact TG molecules and recalculated ratio of particular FA in fat bodies of *B. terrestris* queens in different life phases (relative %).

	Queen's life phase											
	Pharate		Callow		Before hibernation		After hibernation		Egg-laying		Senescent	
FA	Mean	sd	Mean	sd	Mean	sd	Mean	sd	Mean	sd	Mean	sd
12:0	2.20	0.24	1.45	0.42	0.12	0.08	0.32	0.10	0.57	0.48	1.44	1.13
12:1	0.13	0.03	0.00	0.00	0.00	0.00	0.00	0.00	0.01	0.01	0.04	0.04
14:0	10.68	1.51	8.87	0.25	1.83	0.80	3.32	0.81	2.99	2.04	7.40	5.17
14:1	1.89	0.19	0.63	0.26	0.45	0.14	0.10	0.03	0.03	0.05	1.10	0.53
16:0	17.50	1.66	24.07	1.30	11.15	1.55	15.73	0.67	15.68	0.86	19.94	1.56
16:1	11.79	0.30	7.14	0.74	19.42	4.48	18.90	1.44	6.50	0.54	7.84	2.55
18:0	1.96	0.70	3.08	0.52	0.41	0.13	1.03	0.55	3.50	1.49	3.16	1.08
18:1	33.69	1.64	40.33	0.92	57.03	6.46	52.74	2.32	53.31	4.76	41.55	7.57
18:2	6.33	0.93	3.97	0.90	5.82	0.90	2.39	0.21	5.73	1.74	5.32	1.04
18:3	7.31	1.18	4.30	0.90	3.06	0.60	2.80	0.24	7.44	1.41	6.66	1.84
20:1	0.00	0.00	0.00	0.00	0.22	0.10	0.06	0.01	0.00	0.00	0.00	0.00
20:3	0.00	0.00	0.00	0.00	0.10	0.04	0.09	0.02	0.00	0.00	0.14	0.12

sd = standard deviation
