# Supplementary material for: Seasonal Dynamics in the Chemistry and Structure of the Fat Bodies of Bumblebee Queens
Source: PLoS One. 2015 Nov 11;10(11):e0142261. doi: 10.1371/journal.pone.0142261 (PMC4641598; doi:10.1371/journal.pone.0142261)
Supplement: S6 Table — PE, phosphatidylethanolamine; PC, phosphatidylcholine; PS, phosphatidylserine. (PDF) [file pone.0142261.s012.pdf]

S6 Table. The phospholipid membrane composition of fat body of *B. terrestris* queens before and during hibernation obtained by HPLC/MS-ESI technique (relative % in phospholipid fraction; n = 5). PE, phosphatidylethanolamine; PC, phosphatidylcholine; PS, phosphatidylserine.

	Before hibernation		During hibernation	
Substance	mean	std	mean	std
Lyso PE 18:3	0.09	0.08	0.20	0.15
Lyso PE 18:2	0.14	0.17	0.32	0.35
Lyso PE 18:1	1.09	1.08	2.16	2.18
Lyso PC 16:0	0.67	1.08	0.78	0.82
Lyso PC 18:2	0.20	0.17	0.79	0.96
Lyso PC 18:1	2.41	3.72	6.51	6.24
PE 16:1/16:1	0.03	0.03	0.06	0.08
PE 16:0/16:1	0.19	0.15	0.15	0.19
PE 16:1/18:2	0.08	0.08	0.11	0.08
PE 16:0/18:3	0.01	0.01	0.00	0.00
PE 16:1/18:1	0.83	0.45	0.46	0.42
PE 16:0/18:1	2.16	0.75	2.56	0.75
PE 17:0/18:3	0.99	0.40	1.36	0.88
PE 17:0/18:2	0.18	0.21	0.35	0.38
PE 17:1/18:1	0.41	0.21	0.38	0.41
PE 17:0/18:1	0.31	0.20	0.27	0.19
PE 18:3/18:3	0.07	0.06	0.01	0.02
PE 18:3/18:2	0.04	0.07	0.01	0.03
PE 18:3/18:1	1.87	1.34	1.46	0.49
PE 18:2/18:1	0.37	0.34	0.43	0.23
PE 18:3/18:0	0.61	0.39	0.50	0.15
PE 18:1/18:1	15.48	3.01	9.88	4.02
PE 18:2/18:0	0.09	0.07	0.36	0.18
PE 18:1/18:0	2.79	0.99	2.15	0.53
PC 16:1/16:1	0.33	0.09	0.12	0.09
PC 16:1/18:3	0.13	0.13	0.05	0.03
PC 16:0/18:3	0.06	0.08	0.01	0.01
PC 16:1/18:1	6.41	1.37	3.20	0.99
PC 16:0/18:1	9.50	1.15	6.12	1.49
PC 18:3/18:3	0.59	0.78	0.10	0.08
PC 18:2/18:3	0.27	0.32	0.27	0.14
PC 18:1/18:3	6.03	2.41	4.18	1.02
PC 18:1/18:2	3.18	0.46	5.78	1.85
PC 18:0/18:3	0.29	0.18	1.19	1.29
PC 18:1/18:1	38.22	4.91	42.70	7.49
PC 18:0/18:1	2.03	0.39	3.06	1.25
PS 18:2/18:2	0.05	0.05	0.09	0.05
PS 18:0/18:3	0.27	0.23	0.10	0.12
PS 18:1/18:1	0.91	0.29	1.31	0.58
PS 18:0/18:1	0.27	0.30	0.22	0.18
